# Alternative Strategies to Generate Class Activation Maps Supporting AI-based Advice in Vertebral Fracture Detection in X-ray Images

**DOI:** 10.1055/a-2562-2163

**Published:** 2025-06-03

**Authors:** Samuele Pe, Lorenzo Famiglini, Enrico Gallazzi, Chandra Bortolotto, Luisa Carone, Andrea Cisarri, Alberto Salina, Lorenzo Preda, Riccardo Bellazzi, Federico Cabitza, Enea Parimbelli

**Affiliations:** 1 Department of Electrical, Computer and Biomedical Engineering, University of Pavia, Pavia, Italy; 2 Department of Informatics, Systems and Communication, University of Milano-Bicocca, Milan, Italy; 3 ASST G. Pini – CTO, Milan, Italy; 4 Unit of Radiology, Department of Clinical, Surgical, Diagnostic, and Pediatric Sciences, University of Pavia, Pavia, Italy; 5 Department of Radiology, I.R.C.C.S. Policlinic San Matteo Foundation, Pavia, Italy; 6 Department of Reconstructive Surgery and Osteo-articular Infections C.R.I.O. Unit, I.R.C.C.S. Galeazzi Orthopaedic Institute, Milan, Italy; 7 Telfer School of Management, University of Ottawa, Ottawa, Ontario, Canada

**Keywords:** eXplainable artificial intelligence, class activation map, clinical decision support systems, medical imaging, radiology

## Abstract

**Background:**

Balancing artificial intelligence (AI) support with appropriate human oversight is challenging, with associated risks such as *algorithm aversion* and *technology dominance* . Research areas like eXplainable AI (XAI) and Frictional AI aim to address these challenges. Studies have shown that presenting XAI explanations as “juxtaposed evidence” supporting contrasting classifications, rather than just providing predictions, can be beneficial.

**Objectives:**

This study aimed to design and compare multiple pipelines for generating juxtaposed evidence in the form of class activation maps (CAMs) that highlight areas of interest in a fracture detection task with X-ray images.

**Materials and Methods:**

We designed three pipelines to generate such evidence. The pipelines are based on a fracture detection task from 630 thoraco-lumbar X-ray images (48% of which contained fractures). The first, a *single-model* approach, uses an algorithm of the Grad-CAM family applied to a ResNeXt-50 network trained through transfer learning. The second, a *dual-model* approach, employs two networks—one optimized for sensitivity and the other for specificity—providing targeted explanations for positive and negative cases. The third, a *generative* approach, leverages autoencoders to create activation maps from feature tensors, extracted from the raw images. Each approach produced two versions of activation maps: AM3—as we termed it—which captures fine-grained, low-level features, and AM4, highlighting high-level, aggregated features. We conducted a validation study by comparing the generated maps with binary ground-truth masks derived from a consensus of four clinician annotators, identifying the actual locations of fractures in a subset of positive cases.

**Results:**

HiResCAM proved to be the best performing Grad-CAM variant and was used in both the single- and dual-model strategies. The generative approach demonstrated the greatest overlap with the clinicians' assessments, indicating its ability to align with human expertise.

**Conclusion:**

The results highlight the potential of Judicial AI to enhance diagnostic decision-making and foster a synergistic collaboration between humans and AI.

## Introduction

*Machine learning* (ML) and *deep learning* (DL) algorithms have proven to be highly useful in supporting radiological tasks, where they excel at recognizing structures and anomalies in medical images and can generate quantitative scores to describe them. With advanced neural network architectures 1 and modern training techniques, 2 we can now develop effective classifiers that identify patterns in unstructured data. This makes machine-generated opinions a valuable tool, often serving as a useful starting point or second opinion in clinical evaluations.

Despite these advantages, skepticism around *artificial intelligence* (AI) persists. Humans and machines approach problems in fundamentally different ways. For example, in a classification task, both can reach the correct outcome, but they may focus on entirely different features of the data. In diagnostic imaging, AI may emphasize medically irrelevant parts of an image, leading clinicians to disregard its suggestions—a phenomenon known as *algorithm aversion* . Dietvorst et al. 3 found that people are more inclined to trust humans over machine opinions, especially after witnessing even a few errors from the algorithm. Although AI may make fewer mistakes than humans, its errors are often harder to explain, making them particularly questionable in high-stake fields like healthcare. Given this disparity, there is growing interest in understanding how AI algorithms work to assess their reliability. This has led to the emergence of eXplainable AI (XAI), 4 a research field dedicated to making ML models more interpretable, both in healthcare 5 and elsewhere. 6 Traditional models such as logistic regression and decision trees are transparent to humans improving fairness and trust 7 ; nevertheless, many widely used ML models, e.g., neural networks, often function as “black boxes,” processing inputs into outputs in ways that are still opaque to users.

At the same time, machines can process vast amounts of data quickly and often extract more information than humans. This has led to instances of users following AI suggestions blindly, a behavior referred to as *technology dominance* . 8 Skitka et al 9 observed that users who receive machine-generated suggestions tend to follow them uncritically. As a possible solution to this problem, Cabitza et al 10 introduced the concept of *Frictional AI* , showing how introducing cognitive friction—the insertion of obstacles in the decision-making process—can mitigate this issue and encourage thoughtful decision-making. Current decision support systems typically provide a label or confidence score, but Frictional AI proposes a *judicial protocol* that provides “juxtaposed evidence” for each possible outcome, rather than a simple prediction. By offering both supportive and opposing evidence for a given classification, the judicial protocol mirrors the decision-making process in human judicial systems, where multiple perspectives are considered to ensure fair and balanced decisions. This approach can significantly reduce human overreliance on machines, improve trust in AI systems, and enhance user confidence in clinical settings.

These considerations about algorithm aversion and technology dominance are particularly critical in fields like healthcare, where decisions directly impact patients' quality of life. To address these challenges, our study bridges the gap between human and AI by offering a transparent, visual representation of the features influencing the machine's decisions, serving as a robust decision support tool. Specifically, we explore how contrasting evidence, represented through class activation maps (CAMs) 11 —an XAI technique—can be generated and effectively utilized to enhance AI-driven clinical decision support.

## Objectives

In this study, we extend the work of Famiglini et al, 11 particularly in the context of medical images, where contrasting evidence is presented as CAMs, highlighting the most significant parts of an image that the AI used for classification. By displaying the most persuasive CAMs for both positive and negative prediction, clinicians are compelled to consider evidence from both sides. Our contributions focus on the following objectives:

- **OBJ1:** Explore different algorithms for generating CAMs, building on the classifier and dataset from Cabitza et al 13 to identify the best-suited CAM variant for this scenario.
- **OBJ2:** Implement novel alternative approaches for generating contrasting evidence in judicial protocols and compare their results.
- **OBJ3:** Conduct a validation study with expert radiologists to assess the CAMs and overall methodology.

## Materials and Methods

### Materials

#### Class Activation Maps and Metrics for Their Evaluation

CAMs were introduced by Zhou et al 14 as a method to highlight importance of pixel in classification tasks and generate saliency maps. They proposed a convolutional neural network (CNN) architecture, inspired by models like ResNet, 15 ending with a global average pooling (GAP) layer and a fully connected (FC) layer. The CAMs are produced by combining the activations from the last convolutional layer with the weights that link these activations to the output score for the chosen class. However, a major limitation of this approach is that it requires a specific network architecture. To address this, Selvaraju et al 12 introduced *Gradient-weighted CAM* (Grad-CAM), which generates CAMs by computing the partial derivatives of the score function ( *s* ) with respect to the parameters of a generic convolutional layer ( ***A***_*k*_ ).





Several optimized versions of Grad-CAM have been developed. One of the most notable is HiResCAM, proposed by Draelos and Carin, 16 which enhances Grad-CAM by using a Hadamard product to combine the gradient with the activation tensor for generating the CAMs.



HiResCAM has been shown to perform better than Grad-CAM in domains like medicine. For example, in a more recent work, 17 HiResCAM successfully identified the location of pulmonary anomalies in CT scans, while Grad-CAM focused on irrelevant anatomical regions.

Several metrics exist for evaluating the performance of CAM algorithms, starting with robustness. Chattopadhyay et al 18 introduced metrics like *drop in confidence* and *increase in confidence* , which measure changes in classification confidence after the input image is multiplied by its corresponding CAM using a Hadamard product. Rong et al 19 proposed the *Remove and Debias* (ROAD) method, which perturbs both the most and least informative parts of the image and measures the subsequent change in confidence. Unlike previous metrics, ROAD replaces selected pixels with a weighted average of neighboring pixels before evaluation, instead of completely obliterating them.

Another crucial aspect of evaluating CAM algorithms is the sanity check, where the algorithm's output is compared with a reference (often a poor-performing algorithm). Tomsett et al 20 suggested using the result of a Sobel filter as a reference, though other techniques also exist.

#### Dataset

Our data were obtained from the healthcare institute ASST Gaetano Pini – CTO of Milan, Italy, and consisted of X-ray images collected between 2010 and 2020 from 151 patients, all over 18 years old, who had experienced traumatic events. The images were cropped into 630 pictures, each focusing on one or more thoraco-lumbar vertebrae. Of these images, 48% show fractures and the presence or absence of fractures was confirmed by three experienced spine surgeons, using CT and MRI scans for additional verification. The dataset was split into a training set (80%), and validation and test sets (10% each). The study has been approved by the ethical review board Lombardia 6, at Policlinic San Matteo Foundation (Pavia, Italy).

**Fig. 1:**
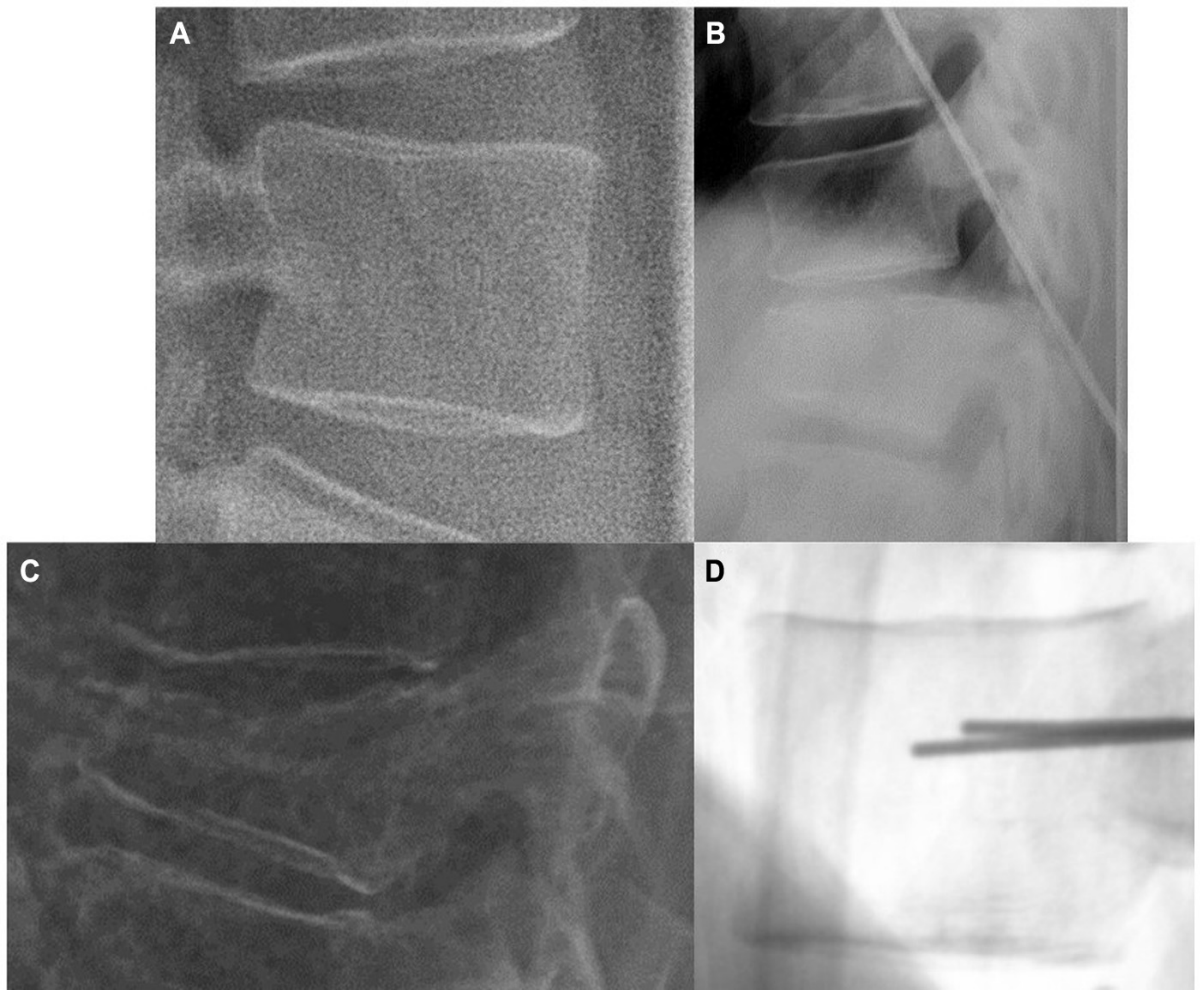
Example of images from the training set, showing images with different scales ( **A** , **B** ), a quite blurry image ( **C** ), and an image of a vertebra presenting both a peculiar coloring scheme and a shadow of an unknown object ( **D** ).

In AI, both the quantity and quality of data are crucial for achieving good results. Although there is no definitive way to determine whether our dataset size is sufficient, a qualitative inspection reveals several issues ( Fig. 1) . The images exhibit variability in coloring schemes and scale; moreover, some images focus on a single vertebra while others show multiple vertebrae. Some radiographs are blurred, and others contain text or foreign objects obscuring parts of the vertebral body.

### Methods

#### Alternative Approaches for the Generation of CAMs

**Fig. 2:**
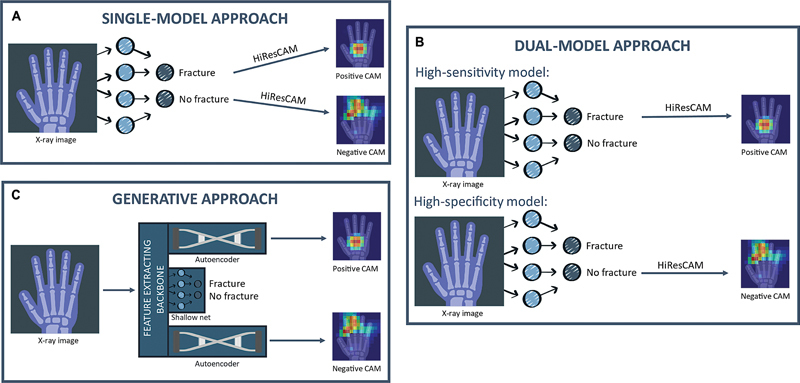
Schematic representation of the proposed approaches for the generation of class activation maps (CAMs). The single-model approach ( **A** ), the dual-model approach ( **B** ), and the generative approach ( **C** ).

We developed three approaches to generate CAMs (OBJ2). The first approach, the *single-model* approach ( Fig. 2A) , involves deploying a ResNeXt-50 network 21 for classification and using a Grad-CAM variant to produce the activation maps. In earlier works, 13 the classification model was pre-trained on ImageNet to handle the small dataset size, and before fine-tuning, the last fully connected (FC) layer was replaced with a simple two-neuron layer. By applying the CAM algorithm to both output neurons, it was used to generate positive and negative maps, corresponding to the “fracture” and “absence of fracture” classes. In the present work, under OBJ1, we experimented with several CAM algorithms [Section 4.1, The single-model approach], selecting the best one for this scenario using quantitative metrics [section 3.1.1, Class Activation Maps and metrics for their evaluation] computed on the validation set. Finally, we completed the pipeline employing the chosen algorithm to generate two sets of activation maps: one from the output of the third ResNeXt block (AM3), representing fine-grained, low-level features, and the other (AM4) highlighting high-level, aggregated features.

The *dual-model* approach ( Fig. 2B) utilizes two CNN classifiers trained similarly to the ResNeXt-50 network: the first model is optimized for sensitivity on the validation set, while the second is optimized for specificity. The training parameters for the two networks are detailed in Table 1 . This configuration allows the first model to specialize in detecting positive instances and recognizing their distinctive features, while the second model focuses on identifying patterns specific to negative instances. CAMs for the “fracture” class are obtained from the sensitivity-optimized model, and CAMs for the “absence of fracture” class are derived from the specificity-optimized model. We tested various CAM algorithms and evaluated their performance separately for each model, as there is no straightforward way to extend the evaluation metrics to this more complex CAM creation scheme. As in the single-model approach, applying the selected CAM algorithm to different layers of the models generates both low-level (AM3) and high-level (AM4) activation maps.

**Table 1:** Characterization of training parameters for the models designed in this work

Model	Training epochs	Batch size	Learning rate	Optimizer
**High-sensitivity model**	20	72	0.081 for the last layer, 0.0091 for the last block, the rest is frozen	Adam
**High-specificity model**	20	120	0.011 for the last layer, 0.0001 for the last block, the rest is frozen	SGD
**Positive (AM4) autoencoder**	200	50	0.80	SGD
**Negative (AM4) autoencoder**	150	60	0.70	SGD
**Positive (AM4) autoencoder**	250	80	0.85	SGD
**Negative (AM4) autoencoder**	300	85	0.75	SGD

Note: The upper section (the first two rows) presents the models developed for the dual-model approach, which uses transfer learning techniques applied to ResNeXt-50 networks. The lower section (the last four rows) outlines the parameters for the autoencoders used in the generative approach. SGD refers to stochastic gradient descent.

**Fig. 3:**
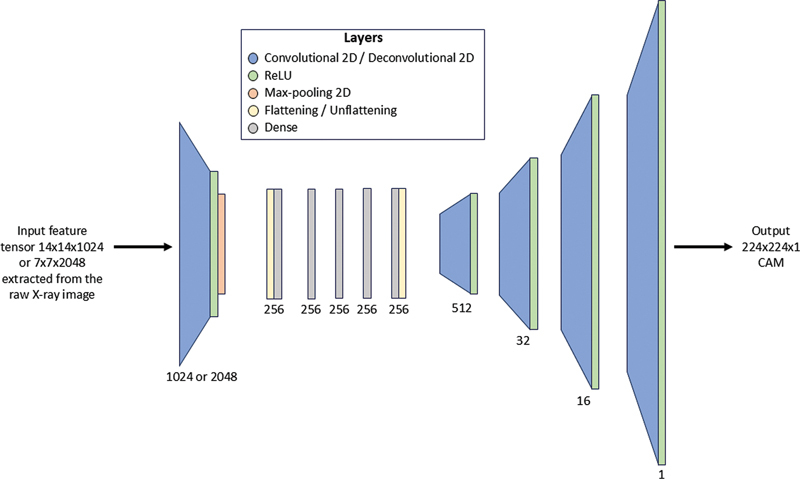
Architecture of an autoencoder model for the generation of class activation maps (CAMs). It takes as input the three-dimensional feature tensors extracted from the raw X-ray image and produces a CAM.

Recognizing that CAM algorithms are designed primarily to explain CNN models and may have limitations in clinical decision support, the *generative* approach ( Fig. 2C) aims to provide a more general solution. This approach uses two autoencoder networks (AE) ( Fig. 3) to generate the positive and negative CAMs, trained in a supervised manner to replicate the single-model CAMs from features extracted from the raw images; to streamline training, we applied a transfer learning (TL) technique using the single-model classifier 13 as a feature extractor. As with the previous approaches, we developed separate models for generating AM3 and AM4 maps, using features taken at different levels of the feature extractor. The parameters used to train these networks are shown in Table 1 .

#### The Validation Study

To compare the results of the three approaches and assess the alignment between human and AI interpretations, we conducted a validation study (OBJ3) with four clinicians (radiologists) from the Policlinic San Matteo Foundation in Pavia, Italy. The participants had varying levels of expertise: two experienced, board-certified radiologists, and two radiology residents. They were selected based on their extensive experience in interpreting X-ray images and diagnosing vertebral fractures. They were recruited based on their availability and willingness to participate in the study.

**Fig. 4:**
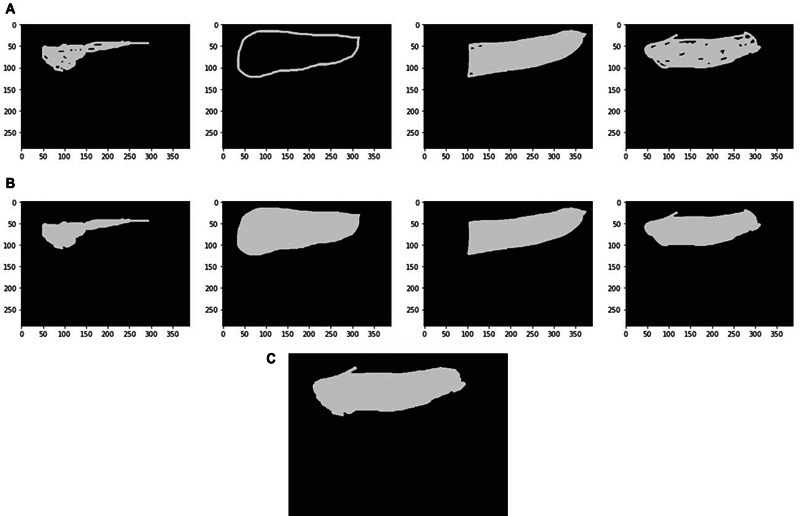
Ground-truth mask creation. We show the masks traced by each clinician ( **A** ), the preprocessing with a fill-holes filter ( **B** ), and the output of STAPLE ( **C** ) merging the four opinions and creating our reference ground truth.

We developed an online tool that displays the 27 positive-labeled images from the test set and allows the clinicians to mark the regions where they believe the fracture is located. After preprocessing the resulting masks using a fill-holes filter and combining them through the STAPLE algorithm, 22 we obtained a ground-truth (GT) binary mask indicating the fracture location. Fig. 4 illustrates the preprocessing steps and the outcome of applying STAPLE to a batch of masks.

We evaluated the overlap between the GT masks and the CAMs generated by each of the three proposed approaches using the Intersection over Union (IoU) and Intersection over Ground Truth (IoGT) indices, defined as follows:





The IoGT metric (of our own definition) represents the proportion of the GT mask that is identified by a given CAM. To calculate these scores, each CAM was binarized through a simple thresholding process based on the mean intensity value of the image and its standard deviation ( *µ* + *σ* ).

Given that we had multiple annotators with varying levels of expertise—two experienced radiologists and two residents—we also conducted an inter-annotator agreement (IAA) evaluation 23 on the binary masks collected. The four masks were compared using *Fleiss' kappa* 24 to assess the overall agreement among the annotators. Additionally, the variance between the specialists' and residents' annotations was evaluated with *Cohen's kappa* , 25 which measures binary IAA. To perform Cohen's binary evaluation, we generated two separate STAPLE combinations for the specialists' and residents' masks.

## Results

### The Single-model Approach

**Fig. 5:**
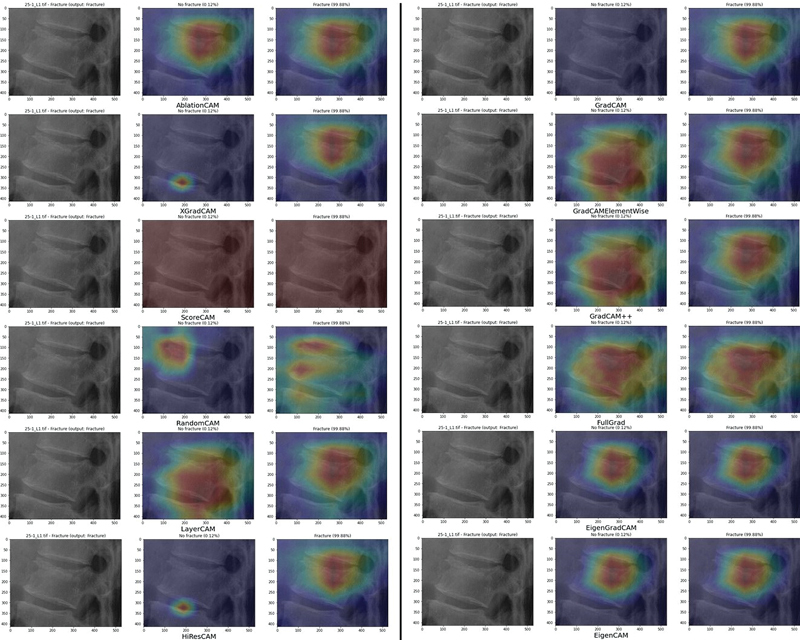
Comparison of the results of different class activation map (CAM) variants associated with the single-model classifier, applied to an image of the validation set.

The neural classifier achieved a training accuracy of 91.84%, with validation and test accuracies of 84.74 and 76.92%, respectively. Before designing this first approach, to determine the most suitable CAM generation algorithm in the context of OBJ1, we evaluated several variants described in the literature, including Grad-CAM, 11 HiResCAM, 16 Grad-CAM + +, 18 XGrad-CAM, 26 and LayerCAM. 27 Other popular algorithms, such as Ablation-CAM, 28 Score-CAM, 29 Eigen-CAM, 30 FullGrad, 31 and DFF, 32 were excluded due to their computational cost and their performance being comparable to more efficient algorithms ( Fig. 5) . The performance of each CAM algorithm is summarized in Table 2 . The first two metrics (drop in confidence and increase in confidence) did not significantly distinguish between the algorithms, as they produced similar values across cases. However, the ROAD metric demonstrated a broader range of variation, showing how each algorithm outperformed the reference sanity-check method (RandomCAM). The best-performing methods, such as Grad-CAM and HiResCAM, were the same ones producing highly distinct maps between positive and negative cases, offering valuable contrasting evidence, as illustrated in Fig. 5 . Based on these quantitative results and existing evidence from the literature, 16 17 we selected HiResCAM for generating CAMs.

**Table 2:** Performance of CAM algorithms applied to the classifier trained for the single-model approach

Algorithm	Drop in confidence	Increase in confidence	ROAD
**GradCAM**	**0.354**	**0.391**	0.141
**HiResCAM**	**0.354**	**0.391**	**0.143**
**Grad-CAM** **+** **+**	0.426	0.326	0.055
**XGrad-CAM**	**0.354**	**0.391**	0.142
**LayerCAM**	0.419	0.326	0.057
**RandomCAM**	0.383	**0.391**	0.005

Abbreviations: CAM, class activation map; ROAD, Remove and Debias.

Note: The best results for each column are shown in bold and also underlined.

**Fig. 6:**
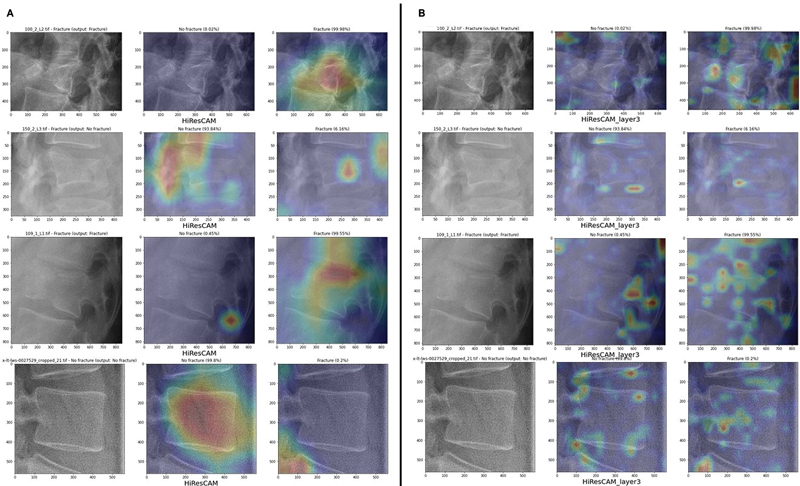
Results for the single-model approach: AM4 ( **A** ) and AM3 ( **B** ) maps for the same images of the training set.

A notable difference between AM4 and AM3 maps ( Fig. 6) is that AM3 maps highlight multiple small regions across the image, whereas AM4 maps focus on fewer, broader areas. When observing AM4 maps ( Fig. 6A) , we see that the size of the highlighted region correlates with the classifier's confidence level, and the positive and negative CAMs do not overlap. In contrast, AM3 maps ( Fig. 6B) show no clear relationship between model confidence and CAM size, though the non-overlapping nature of the maps remains consistent.

### The Dual-model Approach

The sensitive model selected for this approach achieved a perfect sensitivity of 100.00% on the validation set, though its specificity was relatively low at 52.17% as expected, leading to an overall validation accuracy of 76.09%, like the single-model classifier. Its performance on the training and test sets was 94.24 and 73.08%, respectively. On the other hand, the high-specificity model achieved a validation specificity of 82.61% in the validation set, with a sensitivity of 69.57%, and accuracies of 100.00, 76.09, and 71.15% on the training, validation, and test sets, respectively.

**Fig. 7:**
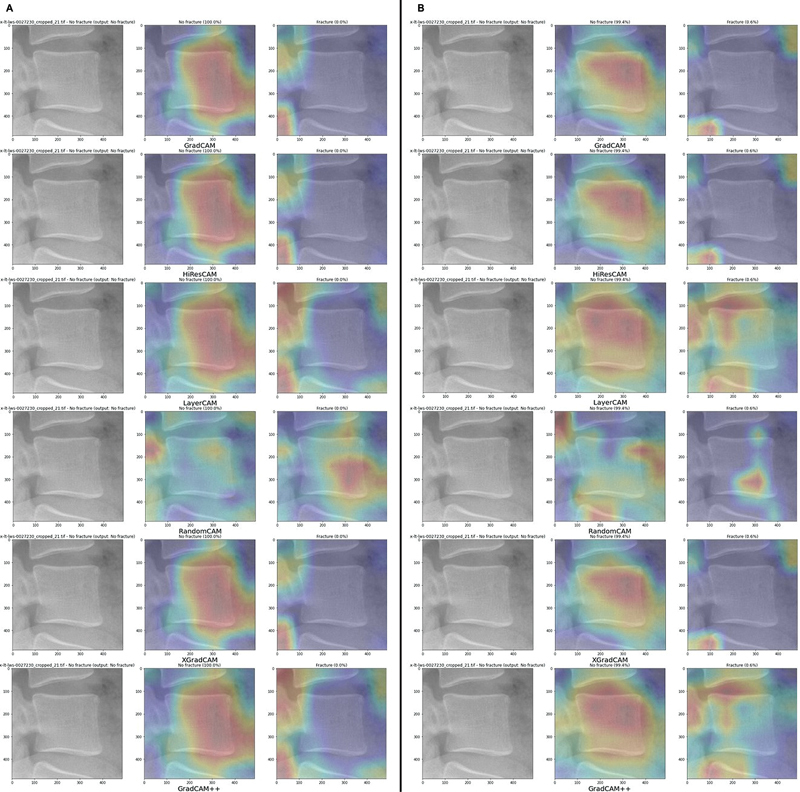
Comparison of the results of different class activation map (CAM) variants associated with the dual-model classifiers: the sensitivity-optimized variant ( **A** ) and the specificity-optimized variant ( **B** ). The algorithms are applied to an image of the validation set.

**Fig. 8:**
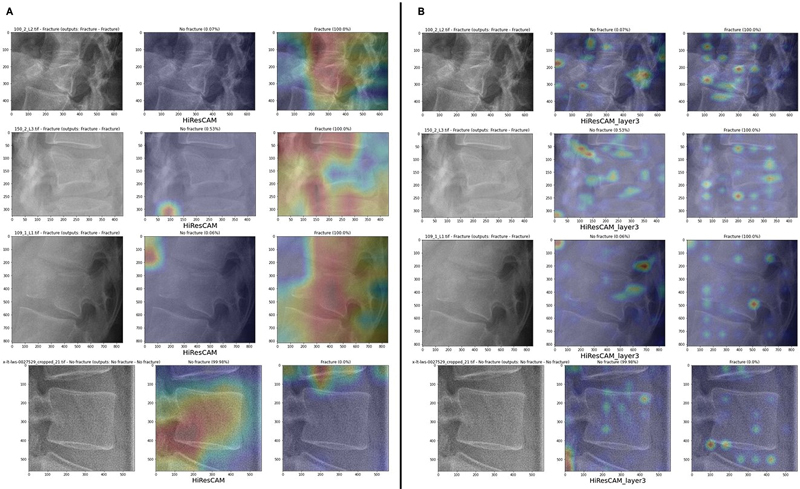
Results for the dual-model approach: AM4 ( **A** ) and AM3 ( **B** ) maps for the same images of the training set.

We applied the same CAM algorithms used in the single-model approach, excluding those with poorer performance, and the results are shown in Fig. 7 . The performance metrics of each CAM algorithm applied to the sensitivity-optimized classifier are presented in Table 3 , while those applied to the specificity-focused classifier are reported in Table 4 . As before, drop in confidence and increase in confidence provided limited help in selecting the best algorithm, so we focused on the ROAD metric. Although HiResCAM was not the top performer from a quantitative perspective, it still ranked among the best algorithms. For consistency with the single-model approach and given its positive features identified in the literature, 17 we decided to continue using HiResCAM. When comparing AM4 and AM3 maps in Fig. 8 , we observed differences and analogies similar to those witnessed for the single-model approach [Section 4.1, The single-model approach].

**Table 3:** Performance of CAM algorithms applied to the sensitivity-optimized classifier trained for the double-model approach

Algorithm	Drop in confidence	Increase in confidence	ROAD
**GradCAM**	**0.173**	**0.565**	0.119
**HiResCAM**	**0.173**	**0.565**	0.107
**Grad-CAM** **+** **+**	0.177	0.370	**0.121**
**XGrad-CAM**	**0.173**	**0.565**	0.118
**LayerCAM**	0.176	0.370	0.111
**RandomCAM**	0.278	0.261	0.002

Abbreviations: CAM, class activation map; ROAD, Remove and Debias.

Note: The best results for each column are shown in bold and are underlined.

**Table 4:** Performance of CAM algorithms applied to the specificity-optimized classifier trained for the double-model approach

Algorithm	Drop in confidence	Increase in confidence	ROAD
**GradCAM**	**0.250**	0.500	**0.092**
**HiResCAM**	**0.250**	0.500	0.090
**Grad-CAM** **+** **+**	0.253	0.413	0.069
**XGrad-CAM**	**0.250**	0.500	0.085
**LayerCAM**	0.264	0.391	0.056
**RandomCAM**	0.364	**0.522**	−0.001

Abbreviations: CAM, class activation map; ROAD, Remove and Debias.

Note: The best results for each column are shown in bold and are underlined.

### The Generative Approach

**Fig. 9:**
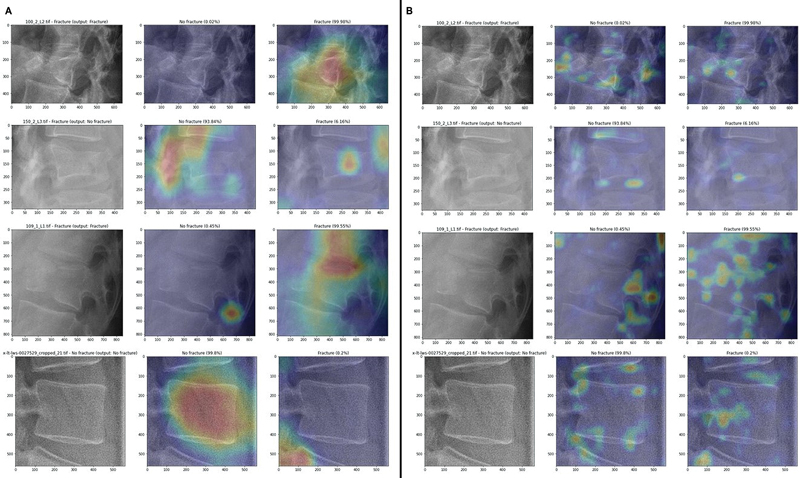
Results for the generative approach: AM4 ( **A** ) and AM3 ( **B** ) maps for the same images of the training set.

We trained four generative models, two for the generation of AM3 and two for AM4. In each case, one model generates the CAMs for the “fracture” class, and the other creates negative “absence of fracture” maps. These models are trained in a supervised manner, with the mean squared errors (MSEs) between the predicted CAM and the one generated by the single-model approach as the loss function. The MSE results are displayed in Table 5 . As suggested by the small errors, there is an almost perfect match between the output CAMs and the single-model maps in the training set ( Fig. 9) . However, this similarity decreases in the validation and test sets, as indicated by the higher MSEs. Despite this, the properties discussed earlier for the CAMs in the single-model approach are still present in the maps created by the generative approach.

**Table 5:** MSEs of the autoencoders trained for the generative approach

Type of CAM produced	Training MSE	Validation MSE	Test MSE
**Positive (AM4)**	3 × 10 ^−5^	8 × 10 ^−4^	1 × 10 ^−3^
**Negative (AM4)**	3 × 10 ^−5^	5 × 10 ^−4^	5 × 10 ^−4^
**Positive (AM3)**	4 × 10 ^−5^	5 × 10 ^−4^	4 × 10 ^−4^
**Negative (AM3)**	2 × 10 ^−5^	5 × 10 ^−4^	5 × 10 ^−4^

Abbreviations: CAM, class activation map; MSE, mean squared error.

### Results of the Validation Study

The inter-annotator agreement (IAA) among the four clinicians shows a low Fleiss' kappa of 0.025, indicating merely a “slight agreement.” This result suggests a notable variability in human judgment regarding the exact location of vertebral fractures in our particular use-case scenario. Comparing the opinions of residents and specialists, the binary Cohen's kappa is 0.030, again reflecting a “slight agreement.” However, given the overall variability already observed, it cannot be concluded that this difference is solely due to disparities in expertise levels.

**Fig. 10:**
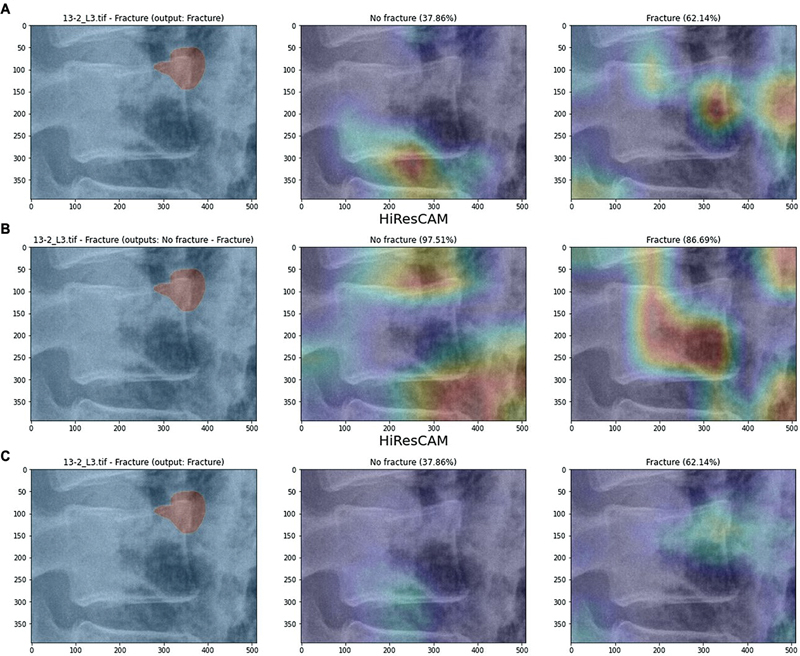
Comparison between the results of the approaches (AM4) and the ground-truth mask for an image in the test set. We display the result of the single-model approach ( **A** ), the dual-model approach ( **B** ), and the generative approach ( **C** ).

**Fig. 11:**
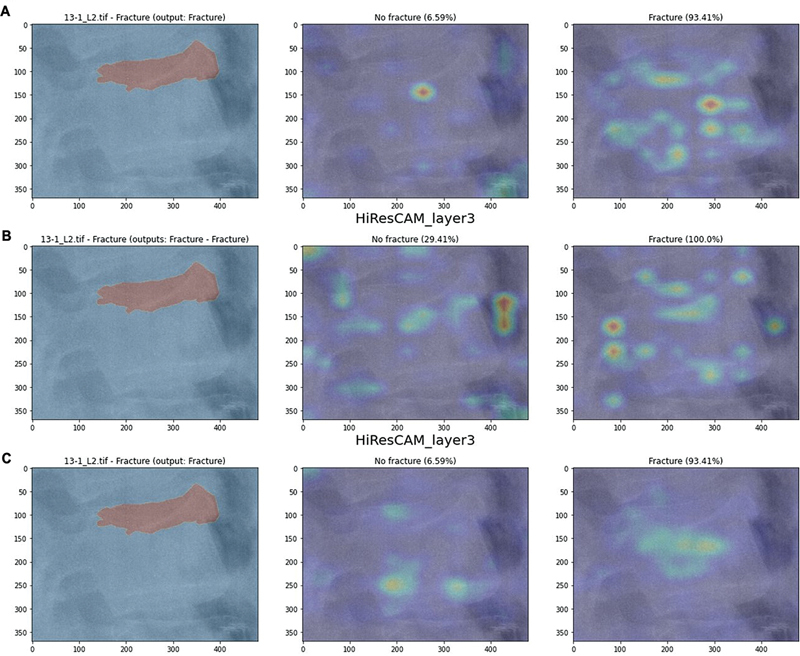
Comparison between the results of the approaches (AM3) and the ground-truth mask for an image in the test set. We display the result of the single-model approach ( **A** ), the dual-model approach ( **B** ), and the generative approach ( **C** ).

When comparing the ground-truth (GT) masks to the CAMs generated by each approach, we expect the positive CAMs to highlight areas near the fracture and the negative CAMs to focus on unrelated regions. Fig. 10 illustrates the results for AM4 compared with the GT masks for an example image, and Fig. 11 provides a similar comparison for AM3 in a different case. The overlap between positive CAMs and GT masks is quantified in Table 6 , and the overlap for the negative CAMs is shown in Table 7 . The generative approach produces positive CAMs with the highest overlap to the GT masks, both for AM4 and, more evidently, for AM3. For negative CAMs, the generative approach performs slightly worse than the other approaches, but the results are still acceptable. Therefore, the generative approach proves to be the most reliable in terms of similarity between the maps and the clinicians' opinions.

**Table 6:** Quantification of the overlapping between positive CAMs and GT masks

Approach	IoU on AM4	IoGT on AM4	IoU on AM3	IoGT on AM3
**Single-model approach**	7.30%	26.84%	3.79%	4.66%
**Dual-model approach**	6.33%	25.11%	1.58%	1.85%
**Generative approach**	**8.34%**	**31.48%**	**7.53%**	**11.62%**

Abbreviations: CAM, class activation map; GT, ground-truth; IoGT, Intersection over Ground Truth; IoU, Intersection over Union.

Note: The best results for each column are shown in bold and are underlined.

**Table 7:** Quantification of overlapping between negative CAMs and GT masks

Approach	IoU on AM4	IoGT on AM4	IoU on AM3	IoGT on AM3
**Single-model approach**	**2.11%**	13.18%	1.95%	2.46%
**Dual-model approach**	2.31%	10.72%	**0.69%**	**0.94%**
**Generative approach**	2.37%	**7.31%**	2.47%	4.60%

Abbreviations: CAM, class activation map; GT, ground-truth; IoGT, Intersection over Ground Truth; IoU, Intersection over Union.

Note: The best results for each column are shown in bold and are underlined.

## Discussion

### Discussion of the Results

To pursue OBJ1, in this study we evaluated different algorithms for generating CAMs from both qualitative and quantitative perspectives. We observed that certain algorithms either failed to produce sufficiently contrasting maps for positive and negative cases or were too time-consuming despite not providing an advantage in terms of performance, leading us to discard them. The remaining algorithms were compared with one another and against a reference (RandomCAM) as part of a sanity check. 20 HiResCAM 16 was selected as the most robust algorithm based on the metrics and literature support. 17

With regards to OBJ2, we designed and implemented three distinct approaches for CAM generation. The first, the single-model approach, applied HiResCAM to a pre-existing neural classifier. 13 The second, the dual-model approach, involved two separate CNNs: one optimized for sensitivity and the other for specificity. By applying HiResCAM to the sensitivity-optimized model, we obtained the positive CAMs and, by applying it to the specificity-optimized model, we produced the negative maps. Lastly, the generative approach used two autoencoders, trained in a supervised manner, to replicate the single-model approach's results, thus generating CAMs directly from features extracted from the raw X-ray images. Each of these approaches produced two sets of CAMs: AM4, which highlighted high-level features, and AM3, which focused on low-level features. Despite the differences between the approaches, some consistent trends were observed, such as the non-overlapping nature of positive and negative maps and the relationship between the extent of AM4 maps and the classification confidence. However, no definitive conclusion can be drawn about which pipeline is the most appropriate for CAM generation: further investigation involving more clinicians, and a larger number of evaluation cases is advisable to get to more definitive conclusions.

Finally, to pursue OBJ3, we designed a validation study involving four clinicians from the Policlinic San Matteo Foundation of Pavia. Their opinions on positive-labeled X-ray images were combined into GT masks indicating the fracture location. These GT masks were then compared with the CAMs created by each approach. The generative method performed best on average, showing the greatest overlap between positive CAMs and GT masks and reduced overlap between the negative CAMs and the GT. The low inter-annotator agreement 23 demonstrated significant variability in clinicians' opinions. On this basis, we infer that a perfect match between human and AI opinions may not be necessary for AI to provide valuable assistance in diagnostic decision-making. AI can play the role of another clinician offering a second opinion, which, although potentially divergent, can still contribute meaningfully to the diagnostic process by presenting alternative insights.

### Limitations

Our work has several limitations. The dataset used is relatively small, and the quality of the X-ray images is at times suboptimal, as discussed in the section Materials. Additionally, the images were cropped during data collection phase (i.e., prior to our study) to focus on one or a few vertebrae only, whereas, in real medical scenarios, clinicians would need to work with radiographs showing wider chunks of the vertebral column. During the validation study, clinicians were also restricted from adjusting the brightness and contrast of the images—adjustments that are commonly available in radiology image visualizers to enhance diagnostic accuracy. Future work should involve using complete radiographs and incorporating improved preprocessing pipelines to assist both the deep learning classifier and the annotators.

Although we proposed three approaches for generating CAMs, we did not include a selection phase to determine the most effective method in terms of diagnostic performance improvement: an ad hoc validation study should be conducted to compare the utility of the CAMs generated by these approaches and, consequently, to evaluate the overall effectiveness of judicial protocols for decision support administration. The current validation study only assessed whether CAMs reflected clinicians' opinions in terms of location of the fracture, but it is essential to identify which method most effectively improves diagnostic accuracy and user confidence.

Future research will focus on designing and implementing additional CAM generation approaches, employing advanced network architectures like U-Nets 33 or attention-based models. 34 Previous results demonstrate the utility of judicial protocols, and this work emphasizes the simplicity and versatility of this methodology. We aim to further explore judicial protocols in various use-case scenarios, including X-ray diagnostics in collaboration with Policlinic San Matteo Foundation and in different healthcare settings, such as neuro-motor rehabilitation.

## Conclusion

This study introduced and evaluated three different approaches for generating CAMs in medical imaging: the single-model, dual-model, and generative approaches. HiResCAM was selected as the best-performing Grad-CAM variant; thus, it was selected for the generation of saliency maps. The generative approach demonstrated superior performance with respect to the other approaches, particularly in aligning with clinicians' opinion. The validation study revealed variability among human experts, which also confirmed that AI-generated CAMs do not need to perfectly match human opinions to be useful in clinical decision-making.

Although promising, the study is limited by a small dataset, image quality issues, and a lack of image preprocessing capabilities. Future work will address these limitations and explore additional CAM generation methods. In addition, judicial protocols will be applied and evaluated in diverse healthcare settings. Ultimately, this study marks the beginning of what we define as Judicial AI, a potential new research direction focused on mitigating existing barriers in the relationship between human and AI and promote their synergic cooperation in critical decision-making, like in healthcare settings.
